# Supplementary figures and images for: wavess: An R package for simulation of adaptive within-host virus sequence evolution
Source: PLoS Comput Biol. 2025 Sep 18;21(9):e1013437. doi: 10.1371/journal.pcbi.1013437 (PMC12459828; doi:10.1371/journal.pcbi.1013437)

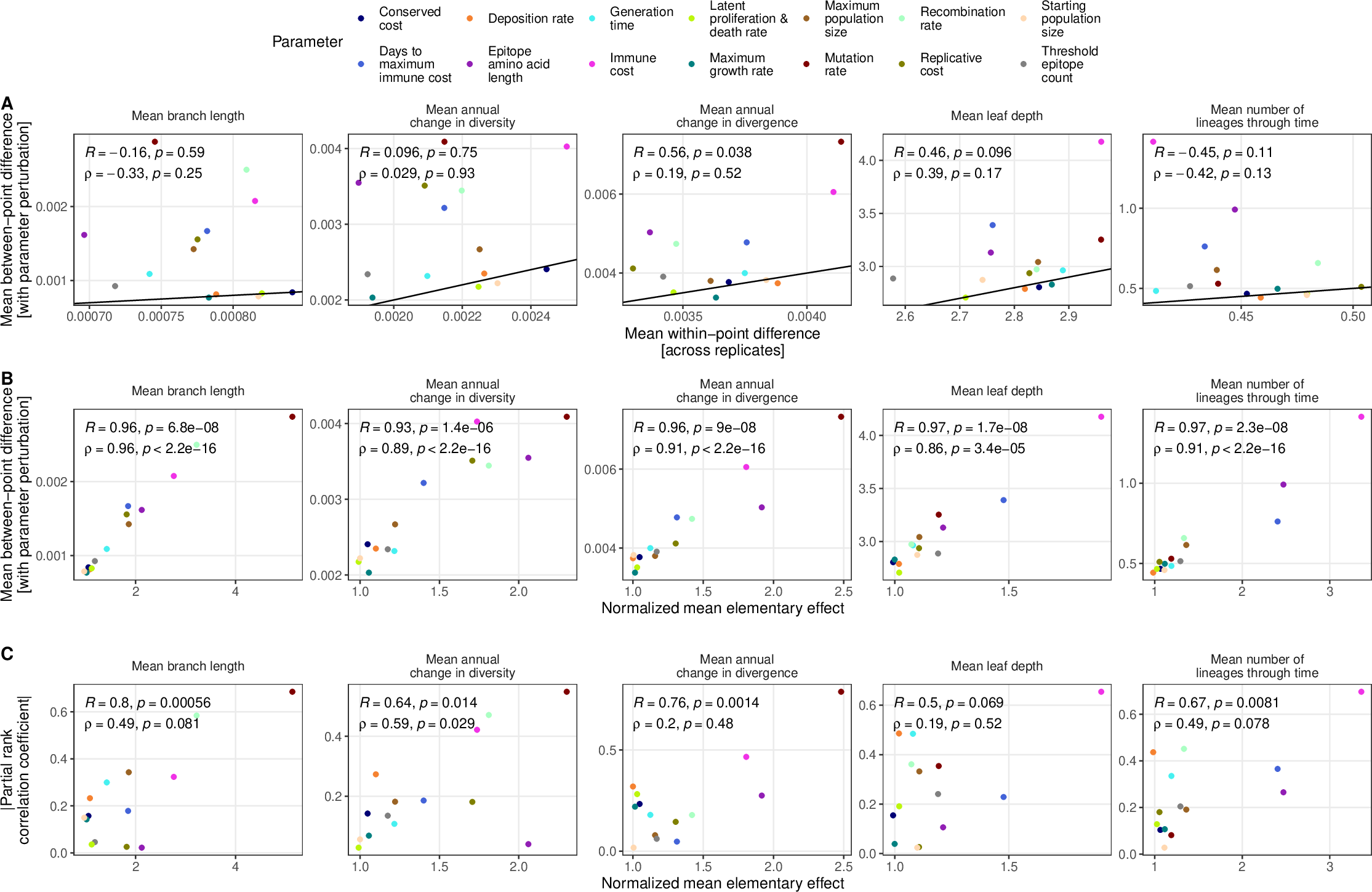

Supplement: S1 Fig — (A) Mean within-point difference in model output across replicates (μ** denominator) compared to mean between-point difference with a parameter perturbation (μ** numerator). Black line is y = x. (B) Normalized mean elementary effect (μ**) compared to mean between-point difference with a parameter perturbation (μ** numerator). (C) Normalized mean elementary effect (μ**) compared to the partial rank correlation coefficient. R indicates Pearson correlation coefficient, ρ indicates Spearman correlation coefficient. (TIF) [file pcbi.1013437.s001.tif]

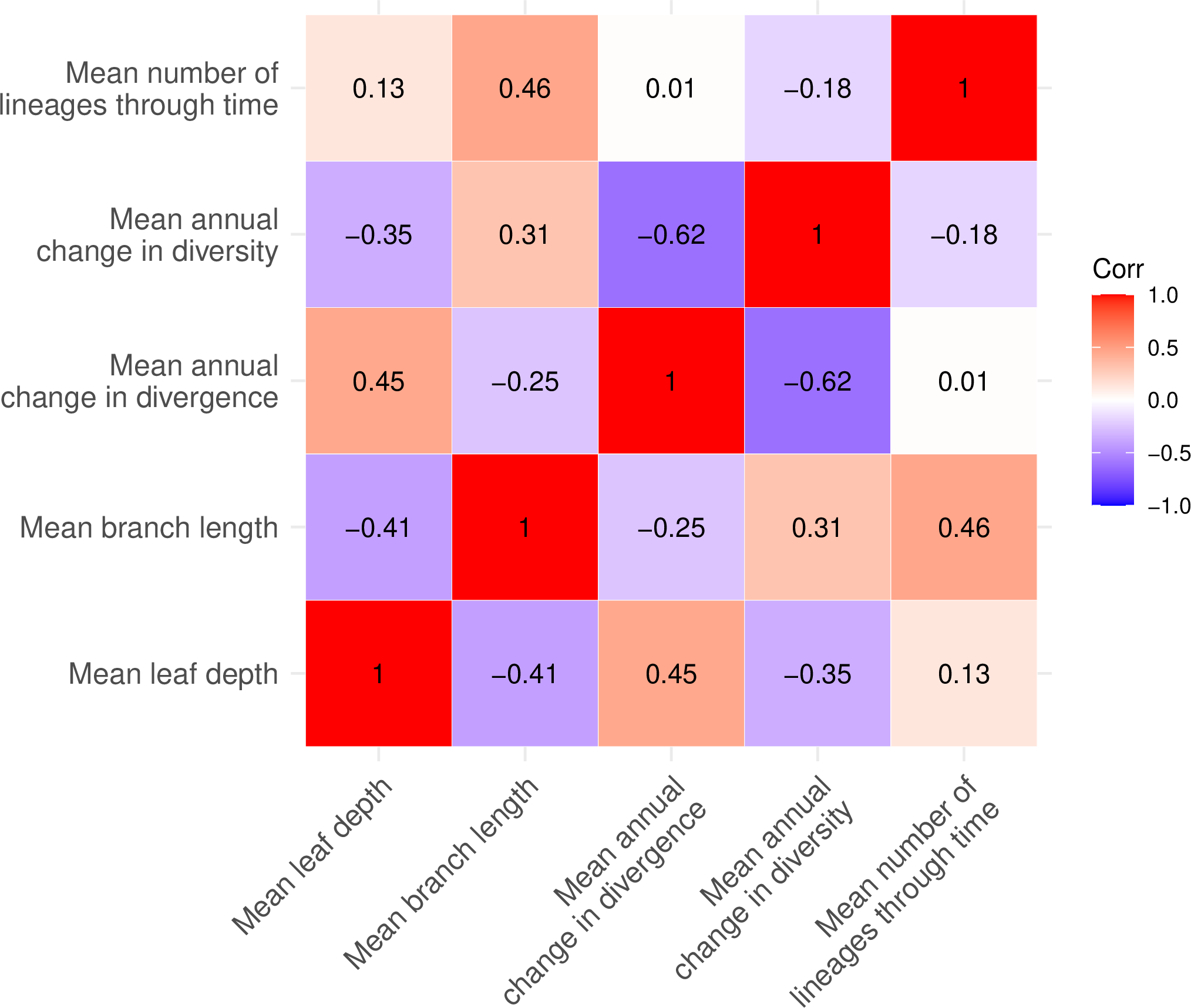

Supplement: S2 Fig — Based on 11 maximum-likelihood phylogenies reconstructed from empirical data. (TIF) [file pcbi.1013437.s002.tif]

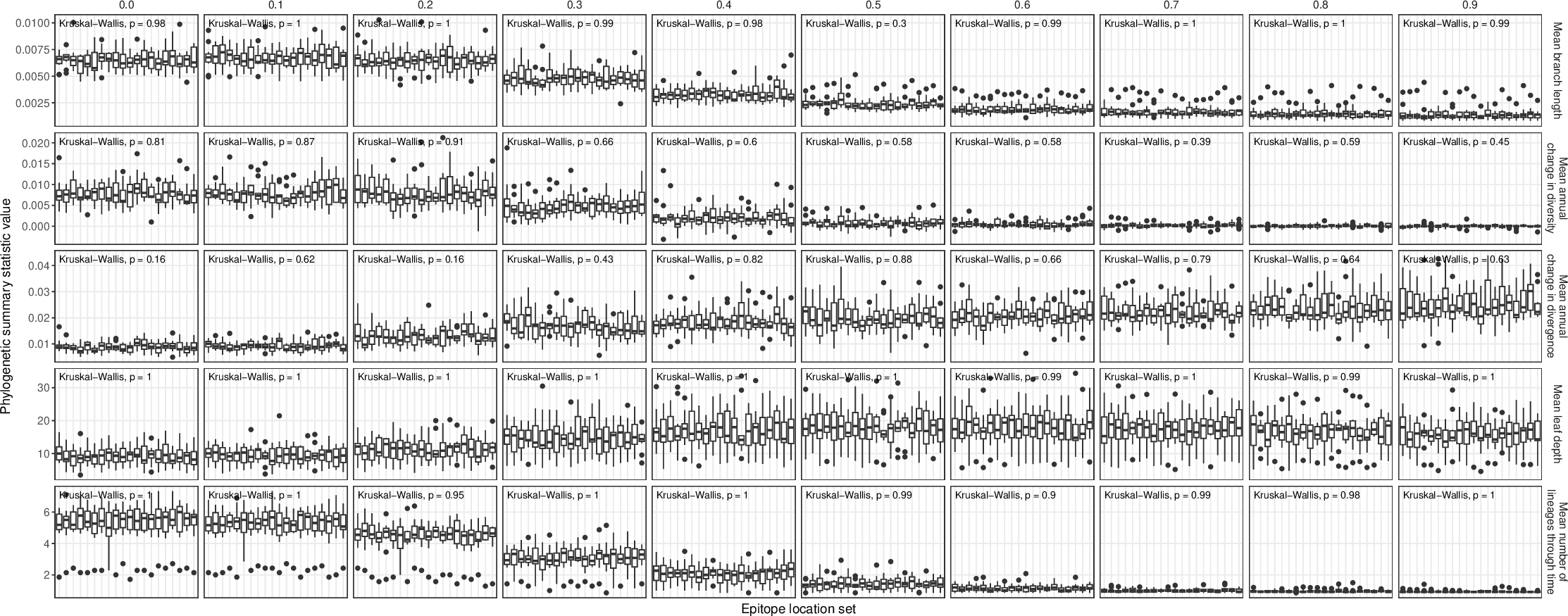

Supplement: S3 Fig — (TIF) [file pcbi.1013437.s003.tif]

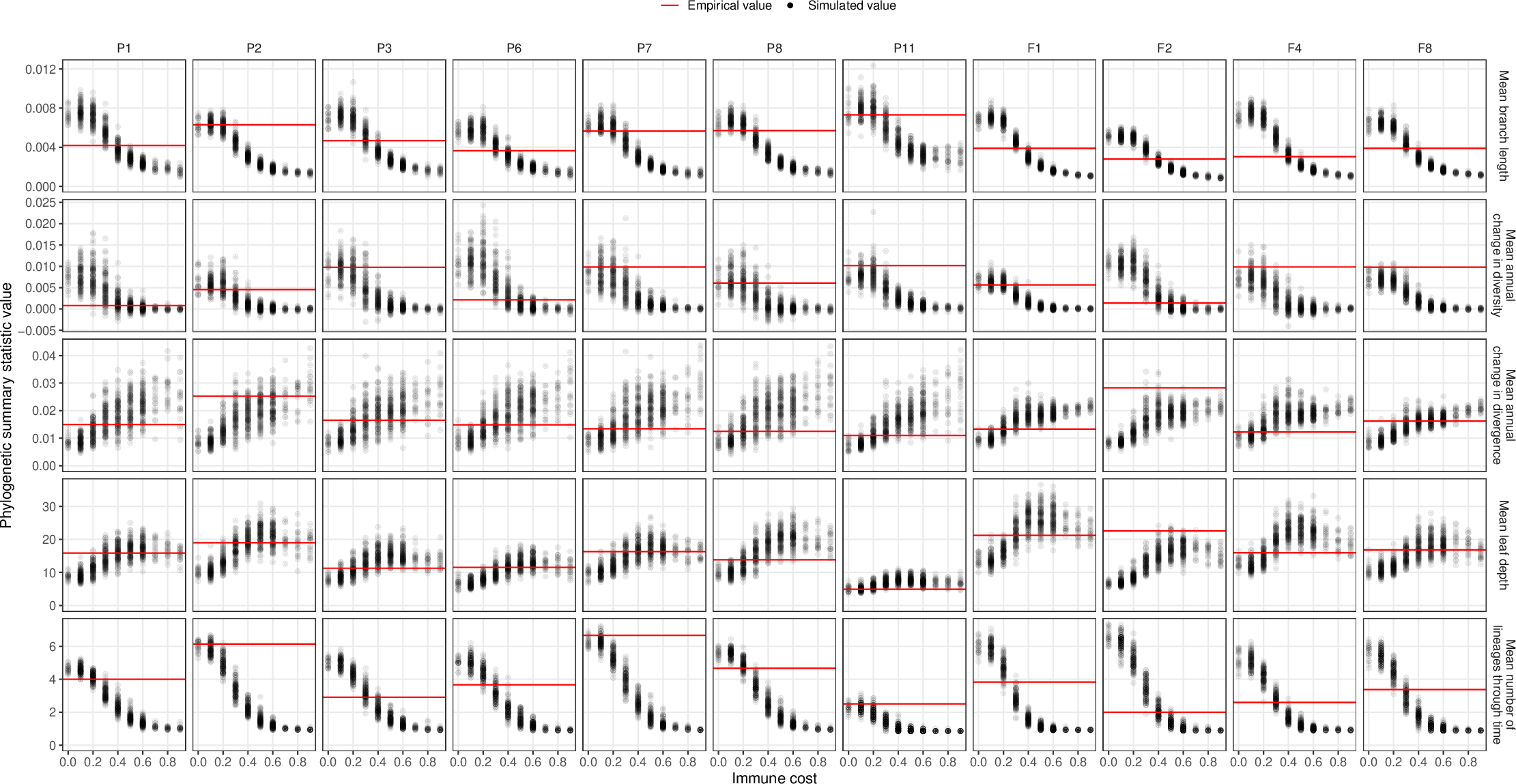

Supplement: S4 Fig — For each real dataset (red line) and simulated dataset across immune costs (black point). (TIF) [file pcbi.1013437.s004.tif]

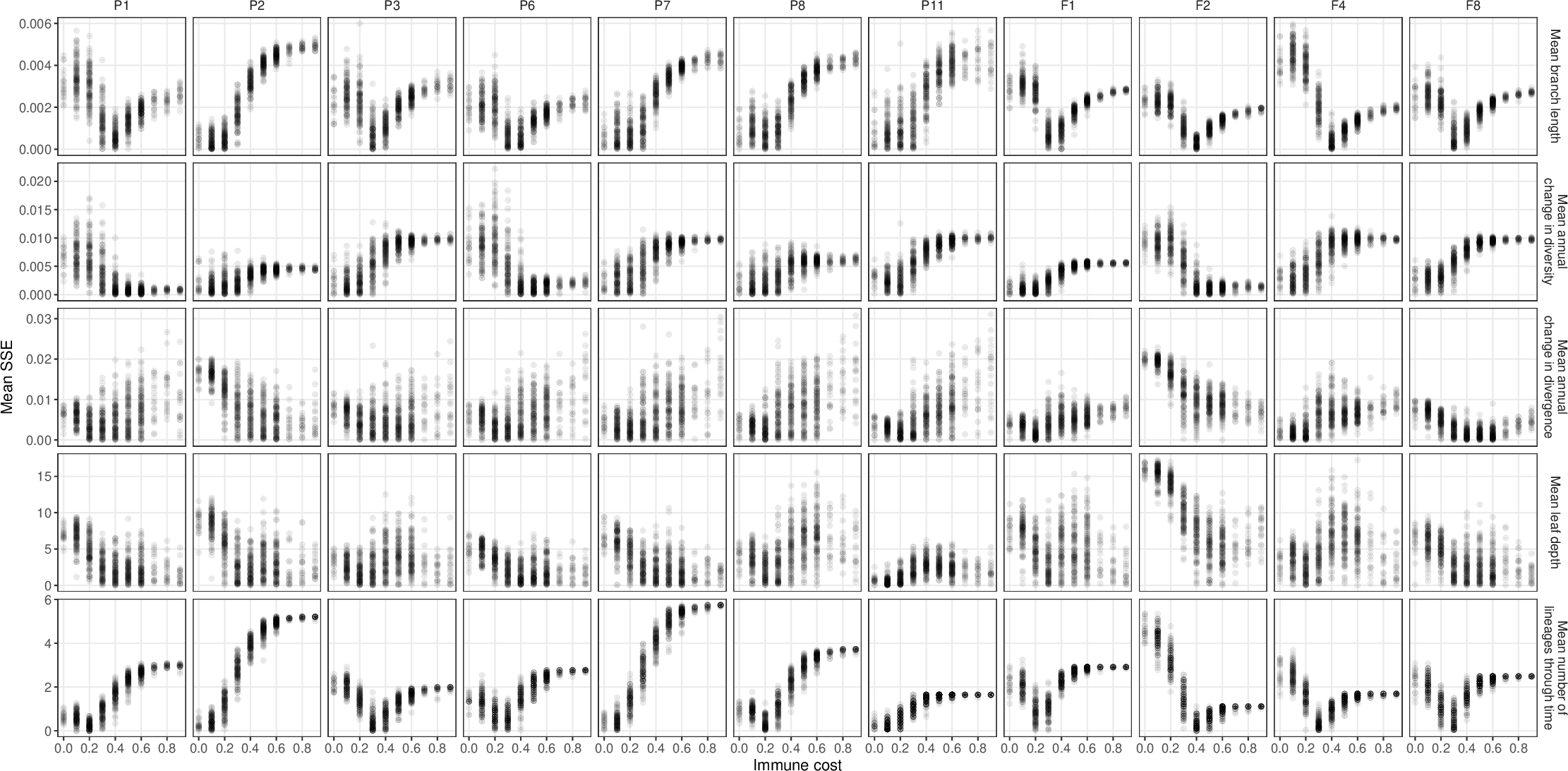

Supplement: S5 Fig — For each dataset and phylogenetic summary statistic across immune costs. (TIF) [file pcbi.1013437.s005.tif]

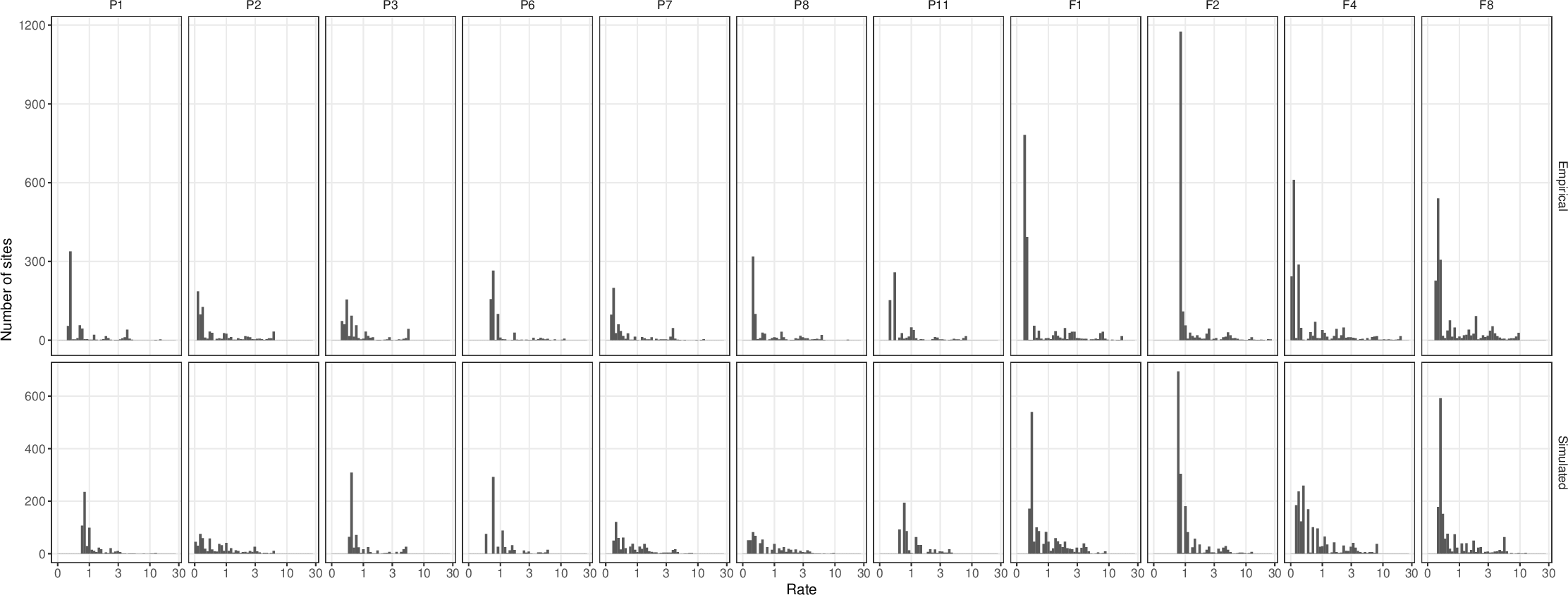

Supplement: S6 Fig — Calculated by IQ-TREE using an empirical Bayesian method where, for each site, the posterior mean site rate across rate categories is weighted by the posterior probability of the site being in each category. The top row is the rates estimated from real sequence alignments and the bottom row is rates estimated from the best-fitting simulated data. (TIF) [file pcbi.1013437.s006.tif]

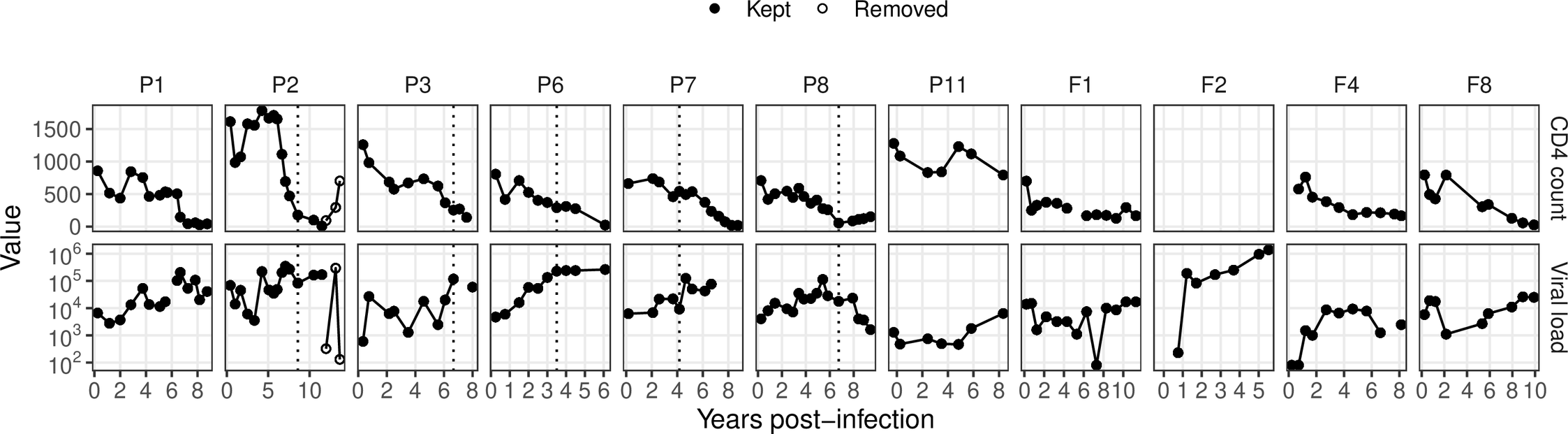

Supplement: S7 Fig — The dotted line indicates when an individual was no longer treatment-naive. Samples removed prior to the analysis are indicated as open circles on the plot. (TIF) [file pcbi.1013437.s007.tif]
