# Supplementary material for: wavess: An R package for simulation of adaptive within-host virus sequence evolution
Source: PLoS Comput Biol. 2025 Sep 18;21(9):e1013437. doi: 10.1371/journal.pcbi.1013437 (PMC12459828; doi:10.1371/journal.pcbi.1013437)
Supplement: Attachment — Submitted filename: wavess_review_responses.pdf [file pcbi.1013437.s008.pdf]

We thank the reviewers for their comments and address their concerns below. Reviewer comments are in gray and our responses are in black. Updates to the text are quoted and underlined.

Reviewer #1: This work offers robust computational tools for simulating virus evolution within a host. Its framework supports recombination, latent infected cell reservoirs, and three types of selection. The system is applied to investigate selection pressures on HIV-1 env sequences, using longitudinal samples from 11 individuals. Validation against real within-host virus data is thorough and rigorous. Overall, this is a well-constructed and relevant contribution.

comments:

(i) Figure 1, insets (G) and (I), from Grenfell (2004; https://doi.org/10.1126/science.1090727) compare HIV population-level phylogeny with within-host HIV phylogeny. In this study, the authors found that the phylogenies reconstructed from the simulated sequences closely resembled the within-host phylogenies derived from empirical data across all tested summary statistics. Given these results, would it be feasible to also reconstruct the population-level HIV phylogeny using this simulation framework?

As wavess was designed as a within-host simulator, it does not have a built-in system to model transmission between individuals. However, it would be feasible to embed wavess within a separate package designed to simulate transmission networks. While it is outside the scope of this manuscript, we have discussed doing exactly this by allowing wavess to be used as a part of SEEPS, another R package developed by our group that models transmission and sampling (`https://github.com/MolEvolEpid/SEEPS`). In this way, within-host evolution, transmission, and sampling could all be modeled at once. We have added this idea and a reference to the SEEPS preprint (recently accepted to Virus Evolution) to the Availability and future directions section of the manuscript:
"Furthermore, `wavess` could be embedded into epidemiological-level models of transmission (e.g. [49]) to make them more realistic."

(ii) Latin Hypercube Sampling (LHS) assumes that the input parameter distributions are uncorrelated. Is this assumption realistic in practice?

This is a good point that we had not seriously considered before. After thinking through the parameters that we included in the sensitivity analysis, we believe that all parameters are either uncorrelated or very weakly correlated. We have explicitly added this assumption to the methods section of the manuscript as well:
"Note that LHS assumes that the input parameter distributions are uncorrelated, which we believe is a reasonable assumption for the parameters used in the sensitivity analysis here."

(iii) second paragraph section 2.3.3: correct "the the".

Fixed.

(iv) section 6.3: The R version v5.3.3 has not yet been released.

Thanks for catching this typo. We have changed it to v4.3.3.

Reviewer #2: In the manuscript "wavess: An R package for agent-based simulation of within-host virus sequence evolution", the authors describe the titular R package and the model underlying it. In addition, they thoroughly test its sensitivity to varying parameters and compare its results to real data. The biological and mathematical theory behind the model as well as its results are convincing, and the manuscript is very clearly written (and so is the model code behind it). The model itself represents a significant contribution to the growing field of agent-based modelling in pathogen evolution.

I tested the R package using the detailed instructions provided by the authors, and it works as advertised. While I am no expert on R, their code appears on closer reading to do what is stated in the

description in the manuscript. The biological assumptions of the model also seem to be well-founded. Finally, the package allows for a large degree of customisation to suit various modelling needs.

My main comments are really more intended as points of discussion or food for thought:

- The authors state that both viruses and their host cells are treated as agents in the model. However, it seems that the infection process itself is not explicitly modelled. Rather, a sample determined through a fitness function is taken from the previous generation of viral sequences and transferred to the following. This is a valid method, but I would not say that it treats the viruses as agents in their own right. As the agency of host cells is also limited, I would almost say that the main agents of the model are the sequences themselves.

After thinking about this a bit more, we have decided that our model is not entirely agent-based, as implied by the reviewer. Rather, we believe that the model is better described as an individual-based model. We have accordingly changed all mention of agent-based models to individual-based models instead. We have also re-worded and re-ordered the Model description sections to remove mention of agents. Instead, we now have the following sections: Infected cells, Viruses (with sub-sections for mutation and recombination), and Selection. Finally, we changed the "a" in `wavess` to stand for "adaptive" instead of "agent-based" (so wavess now stands for within-host adaptive virus evolution sequence simulator). We believe that these changes more accurately describe the model.

- As a consequence of the above, the phylogeny of the model strains cannot be tracked directly, but has to be reconstructed. Again, this is not a problem as such, but one advantage of the agent-based approach can be the ability to directly track relationships between individual agents (e.g., ancestors and descendants). I think a small note on this choice of methodology, possibly in the section on future modelling directions, could benefit the discussion.

Good point. We have discussed adding this to the model, and in fact a much earlier version of the model did track ancestors and descendants. The main issue is that it slows down model runtime a lot. However, we do understand that in some cases this feature could be useful, so we have modified one of our future directions to include this:
"(2) tracking recombination events and virus ancestors and descendants to enable the generation of ancestral recombination graphs and genealogies".

Minor comments:

- Drawing a random number from a beta distribution to calculate cross-reactivity seems reasonable, but it would be a good idea with a note on the reasoning behind using this specific distribution.

We have added this explanation to the text:
"We chose to use a Beta distribution because we assume that larger edit distances between new and recognized epitopes correlate with lower cross-reactivity."
Using a non-squared version of h ($Beta(1, h)$) is equivalent to the product of h iid unit uniform random variables. However, we chose to square the h ($Beta(1, h^2)$) because we thought that the strength of cross-reactivity should decrease faster than when h is not squared.

- The expression given in eq. 3 appears to always give an immune cost of 0 at time t0. However, eq. 4 then describes a starting immune cost. Should this be added to eq. 3?

After re-reading this section, we realized that we did not describe the immune response process as clearly or accurately as we should have. Therefore, we have updated the description substantially, particularly related to how cross-reactivity works. In this process, we clarified how eq. 3 and eq. 4 are related to each other:
"The fitness cost of the cross-reactive epitope remains the same across generations unless it reaches the aforementioned user-defined frequency in the population. If this happens, then the immune response evolves according to Eq 3, where the epitope recognition generation t_i^0 is based on the cross-reactive immune cost and the original time to maximum immune cost: $t - c_{i_x} g$. This is to maintain the same slope in immune cost increase over time for all epitopes at a given location."

- It is stated in section 4.2 that 96 % of empirical summary statistics fall within the range of simulated statistics. Since a probability distribution is calculated based on the model results, would it be possible to test whether this observation matches the theoretical probability of outliers?

After giving this more thought, we realized that the range is not the best summary statistic to compare to, as it is very dependent on the sample size. Instead, we report a different comparison of the empirical to simulated summary statistics:
"Across all 11 individuals, the empirical summary statistics were on average 0.78 standard deviations away from the mean simulated value (range: [0.01, 2.36])."

- In eq. 8, the sum runs over the variable t, but no t appears inside the sum. It would make the equation easier to understand to explicitly show what exactly is summed over.

Good point. We have updated the formula to include the trajectory (what used to be t, which we have now changed to r for clarity since t is often used for time):

$$\mu_k^{**} = \frac{1}{m} \sum_{r=1}^{m} \frac{B_{r_k}}{W_{r_k}}, \tag{1}$$

We have also updated Eq 6 and 7 to reflect this change as well.

- On page 10, line 4: "We next subset the simulations to include...". I found this sentence hard to parse, and suggest replacing it with "We next took a subset of the simulations including..."

We changed the text as suggested.

- Page 12, line 7: a small grammatical error in "...which parameters was perturbed..."

We changed "was" to "were".

All in all, I find this model highly interesting and the associated package quite user-friendly. I thank the authors for their contribution to this interesting field.

Reviewer #3: This paper serves as a comprehensive introduction and explanation of a recently implemented R package for simulating within-host evolution of viral sequences. The package simulates models of viral fitness and selection, host immune response, latent reservoirs, and recombination. The model virus that this was built for is HIV but it is applicable well beyond that.

I found the paper easy to follow and well written. The package works as described and has comprehensive vignettes to walk the user through the main functions.

In my view, the paper is publishable as is but could be improved by addressing the following:

0. I think you should make some of the use cases for this software clearer - how would people use it to "allow for the investigation of factors such as the role of recombination"? (to quote the abstract). An obvious way would be to do parameter inference eg via approximate Bayesian computation or similar - you could mention and reference some works along these lines.

We have updated the intro to include this:
"A realistic model of within-host virus evolution calibrated to real sequence data can overcome some of these limitations and enable us to, for instance, investigate sequence evolution over time under different biological scenarios, or perform parameter inference via approximate Bayesian computation."

1. in the intro, explain how the paper is structured. Many of the details are relegated to section 6 - it would be good to signal that up front.

We have added this sentence to the end of the intro:
"Below, we first describe the model and package implementation, then delve into a theoretical sensitivity analysis followed by comparison of model output to empirical data, and finally provide detailed methods for the theoretical and empirical analyses."

2. "Many modeling frameworks" referred to in the intro but only 2 referenced. Add some references here or point to another article where we can find a fuller list.

We have updated the text and added a reference to a list of 234 genetic data simulators (`https://surveillance.cancer.gov/genetic-simulation-resources/home/`):
"While many genetic simulation software packages exist [1], few are tailored to within-host evolution of viruses. Those that are applicable to virus evolution tend to be individual-based forward simulation models since important biological features such as recombination and selection can be easily incorporated (e.g., the general simulation framework SLiM [2] and the virus-specific simulation framework SANTA-SIM [3])."

3. figure 4 needs to be larger to be able to read it, currently lots of detail and a very small font

Thank you for pointing this out. We have increased the text size in the figure.

4. Section 3.2 Page 8: I was confused by "Mean number of transitions per timepoint" as I was unsure what you meant by transition (my mind first turned to transitions vs transversions.) Looking back several pages in the paper I understood but you could remind readers here what you mean.

We agree that this wording is confusing, so we changed the name of the summary statistic to instead be "mean number of lineages through time" because we think this captures the essence of the summary statistic more clearly. We have modified this in the text, the figures, and the package.

5. IN the R package, the vignettes are not being found by the system. Eg:

> library(wavess)
> vignette("prepare_input_data")
Warning message:
vignette 'prepare_input_data' not found

By default, `devtools::install_github()` does not build vignettes locally. If you'd like to build the vignettes locally, you must use the command `devtools::install_github("MolEvolEpid/wavess", build_vignettes = TRUE)`. We have added this clarification to the README on the GitHub page and the website.

6. (v minor point) In the vignettes, pipes `|>` and `%>%` are both used - choose one (`|>` works throughout by the looks)

Good catch. We changed all of the `%>%` to `|>` to be consistent, except in one instance where `%>%` is needed.

7. With any simulation package, users always ask "but what about...?" In section 5 you discuss a little about future directions but it is not clear how you have structured the implementation to allow others to contribute extensions themselves. THis would be worth a word (or at least thinking about).

We have added a section to the GitHub README describing how to contribute: "You are of course welcome to fork the package for your own use. If you would like for your changes to be incorporated into the latest version of `wavess`, please open an issue related to your proposed change, create a pull request with the implemented change, or reach out to tkl@lanl.gov, nsambaturu@binghamton.edu, or zenalapp@lanl.gov."
